# A child presenting with tuberculous spondylitis in a single third cervical vertebra: a case report

**DOI:** 10.1186/1752-1947-8-284

**Published:** 2014-08-23

**Authors:** Manouri P Senanayake, Irantha Karunaratne

**Affiliations:** 1 Department of Paediatrics, Faculty of Medicine, University of Colombo, Kynsey Road, Colombo 8, Sri Lanka; 2 Lady Ridgeway Children’s Hospital Colombo, Colombo 8, Sri Lanka

**Keywords:** Cervical tuberculosis, Single vertebra disease, Third cervical vertebra

## Abstract

**Introduction:**

Despite a global reduction in tuberculosis, extrapulmonary tuberculosis is increasing. Spinal tuberculosis remains the commonest form of skeletal tuberculosis. Cervical spine involvement is rare but is the most dangerous form because of diagnostic difficulties and serious residual disability. We report a child who had single vertebral involvement of her third cervical vertebra which is extremely rare. To the best of our knowledge isolated third cervical vertebra involvement in a child by tuberculosis has not been reported previously. Difficulties in obtaining material for histology and bacterial culture from this critical location and how the diagnosis was reached despite these challenges are highlighted.

**Case presentation:**

A 10-year-old Sinhalese girl developed painful torticollis and ‘cries during sleep’. She had received Bacillus Calmette–Guérin vaccine at birth, was well nourished, and had no loss of weight, anorexia or contact with tuberculosis. A plain radiograph of her neck showed a collapsed third cervical vertebra with no disc involvement. Magnetic resonance imaging confirmed isolated destruction of third cervical vertebra associated with prevertebral soft tissue swelling indenting the thecal sac without cord compression. Her chest radiograph was normal. There was peripheral lymphocytosis, elevated erythrocyte sedimentation rate, negative tuberculin (Mantoux) test, and negative QuantiFERON®-TB GoldIn-Tube assay. Invasive procedures to obtain tissue for histology, smear or culture were perceived by parents as dangerous due to surrounding critical structures and consent was denied. The differential diagnosis included spinal tuberculosis and unifocal Langerhan cell histiocytosis. Nocturnal symptoms and the prevertebral collection of soft tissue (‘cold abscess’) were characteristic of tuberculosis, and nonspecific findings of elevated erythrocyte sedimentation rate and lymphocytosis supported this diagnosis. An incidental finding of a calcified hepatic nodule when evaluating for multifocal histiocytosis was presumed to be tuberculous because schistosomiasis and visceral leishmaniasis were extremely rare in her country of residence, Sri Lanka. Empirical treatment with a 12-month course of antitubercular therapy resulted in clinical recovery and radiological healing. There was no kyphosis or neurological sequel.

**Conclusions:**

This report highlights to clinicians the value of a high index of suspicion and careful history taking in spinal tuberculosis; and how a combination of nonspecific findings helped reach a clinicoradiological diagnosis.

## Introduction

Cervical spine tuberculosis (TB) is the rarest and most dangerous from of tuberculous spondylitis [1,2]. Although early diagnosis and prompt treatment are essential for the prevention of severe disability, the diagnostic challenges inherent to spinal TB are enhanced when cervical spine is involved. Single vertebra disease which is extremely rare [3] makes the diagnosis even more elusive. This case report describes how a high index of suspicion and a combination of non-specific findings helped in diagnosing TB as the cause of an isolated collapsed third cervical vertebra (C3) in a 10-year-old girl.

## Case presentation

A 10-year-old Bacillus Calmette–Guérin (BCG) vaccinated, Sri Lankan girl presented with painful torticollis for 6 weeks with pain worse at night. There was no preceding trauma to her head or neck. Cries during sleep and restless sleep were frequent occurrences. All her other joints were pain free. There was no fever, sore throat, dysphagia, vertigo, cough, night sweats, anorexia or weight loss. Her past medical history was unremarkable. BCG immunization in neonatal period was followed by normal reaction and scar. Her parents and siblings were healthy. There was no contact with TB.

**Figure 1:**
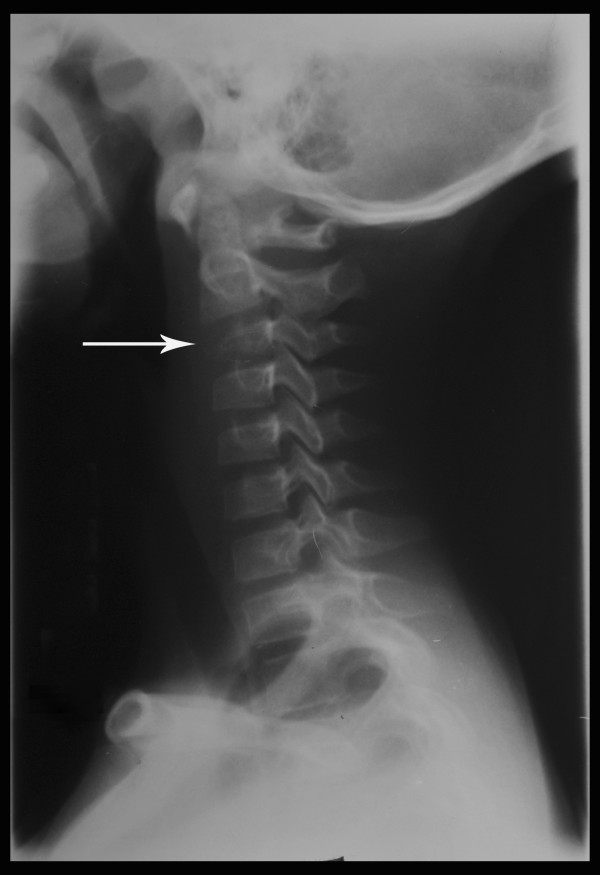
Plain radiograph showing straight spine with destruction and collapse of third cervical vertebra anteriorly (arrow) and normal intervertebral disc spaces.

**Figure 2:**
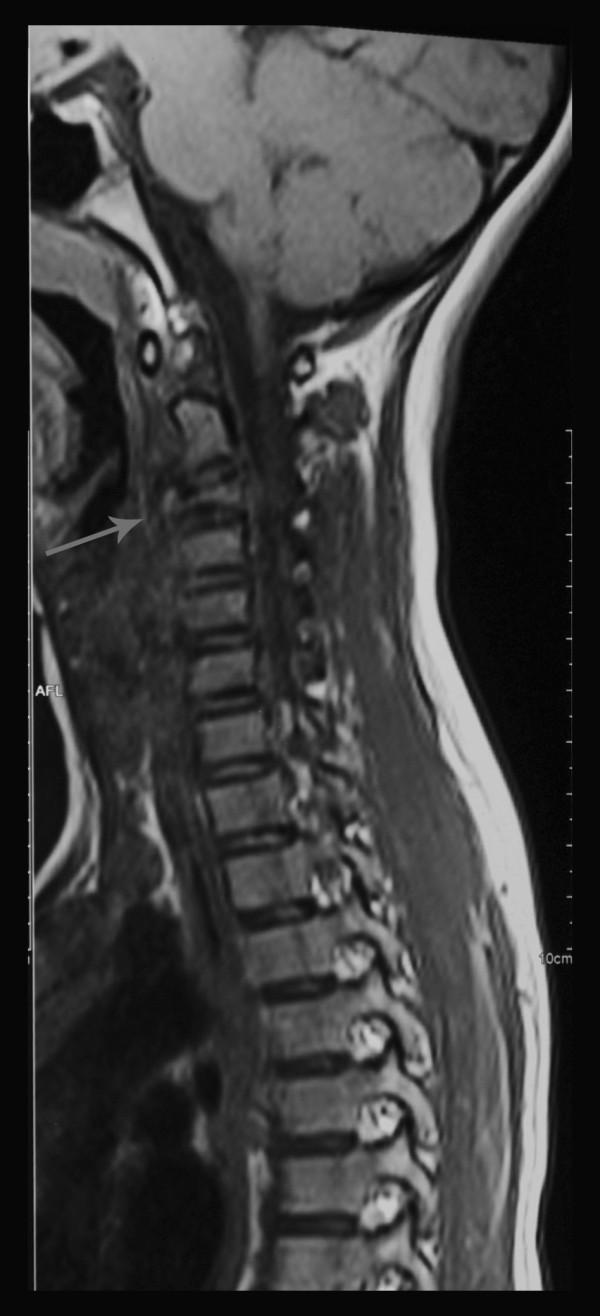
Magnetic resonance imaging showing loss of third cervical vertebra body height, asymmetrical prevertebral soft tissue thickening (cold-abscess) (arrow) and indentation of thecal sac without cord compression.

On physical examination she was adequately nourished (weight and height at 50th centile), systemically well with no lymphadenopathy. All her neck movements were painful and restricted. Muscle spasm and tenderness over her upper cervical spine were present. Kernig’s sign was negative. There was no papilloedema, cranial nerve involvement, muscle wasting or weakness. All tendon reflexes were elicitable normally. The rest of her systems’ examination was normal.Plain radiography showed destruction and collapse of C3 with normal intervertebral disc spaces (Figure 1). A straight cervical spine was attributed to muscle spasm in the acute phase. Kyphosis was not present. Magnetic resonance imaging (MRI) showed loss of vertebral body height in C3, asymmetrical prevertebral soft tissue thickening and indentation of thecal sac without cord compression (Figure 2). Her chest radiograph was normal. The results of a Mantoux test and QuantiFERON TB were negative. Human immunodeficiency virus was excluded on serology. Primary immune deficiency was excluded with normal antibody levels and T cell function.

**Figure 3:**
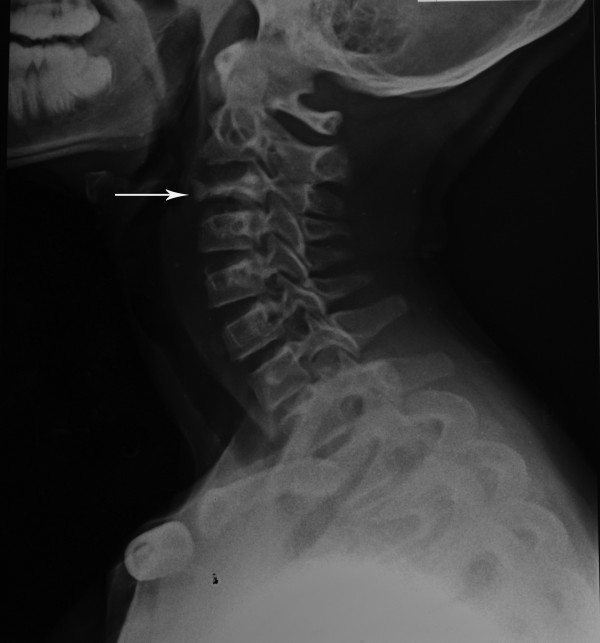
Radiograph taken at end of treatment showing increased height and density of anterior portion of third cervical vertebra (arrow) and normal spine curvature.

Her erythrocyte sedimentation rate (ESR) was 60mm (1st hour), haemoglobin 10.1g/dL, platelets 184×10^9^/L and white cell count 4.0×10^9^ (neutrophils 25%, lymphocytes 72%, monocytes 2%, eosinophils 1%). There were no abnormal cells in her blood picture or bone marrow. Obtaining tissue for histology, smear or culture was not possible because her parents perceived the technically challenging investigative procedures as unsafe and refused consent.A quadruple regimen of antitubercular medication (isoniazid, rifampicin, ethambutol and pyrazinamide) was started empirically with a cervical collar for immobilization. Pain responded in 3 weeks. Radiographic evidence of linear calcification was present after 3 months of treatment confirming the diagnosis of spinal TB. Healing was considered partial and the quadruple regimen was further extended. MRI at 9 months showed increased vertebral body height with resolution of prevertebral swelling. A full course of 12 months antitubercular treatment resulted in anterior portion of third vertebra showing increase in height and density with normal spine curvature (Figure 3). Treatment was discontinued and regular follow up for deformity of spine arranged.

## Discussion

A recent global resurgence of TB resulted in the United Nations’ Millennium Development Goal 6 targeting halving the TB prevalence and mortality rates of 1990 by 2015. Although this target is on track, extra pulmonary disease which is diagnostically more difficult is showing a relative increase [4,5]. The spine is the commonest site of skeletal TB and when cervical vertebrae are involved, the risk of severe disability is high.

The classical clinical picture in spinal TB is local pain, tenderness, stiffness and muscle spasm of several weeks’ duration. Our patient had this presentation but complained of neck pain instead of backache which is the finding in lumbar or thoracic spine TB which are much commoner [6]. Careful history revealed two other symptoms characteristic of spinal TB: ‘cries during sleep’ and ‘restless sleep’. Fever, weight loss and anorexia were lacking in our patient and are absent in the majority of spinal TB patients. Chest radiography was also unhelpful as reported in previous cases of spinal TB [6]. The diagnosis was further obscured by negative tuberculin skin testing and interferon gamma release assay which can both give falsely negative results in children [7].

Demonstration of tubercle bacilli in the skeletal lesion is the gold standard for diagnosis of TB [8]. However, previous reports claim that no series was able to do so in all cases, and empirical treatment is recommended when spinal TB is suspected [9,10].

We were unable to obtain bone biopsy or material for smear and culture due to the difficult trajectory needed for imaging-guided needle biopsy not being permitted by her parents. Surgical intervention was not pursued because false-negative results of biopsy, negative smear for acid-fast bacilli and failure to culture *Mycobacterium tuberculosis* are common.

It is the rich vasculature of cancellous bone of vertebral bodies that causes haematogenous spread of *M. tuberculosis* and tuberculous spondylitis. Arterial supply to vertebrae is from anterior and posterior spinal arteries with segmental arteries bifurcating to supply the upper and lower halves of adjacent vertebrae. This causes the characteristic findings of spinal TB:the destruction of adjacent vertebral bodies and intervertebral disc involvement.

Single vertebral disease without involvement of the disc, as seen in our patient, is very unusual. This has been described in a few adults [10]. The mechanism by which the tubercle bacilli were deposited in a single vertebral body without involving adjacent vertebrae or the disc remains unclear and early stage of disease is the probable explanation. Further, cervical TB is said to occur in only 0.3% to 2.2% of spine TB [6,11,12].

Two conditions considered in differential diagnosis were an infective process or eosinophilic granuloma of bone (unifocal Langerhan cell histiocytosis, LCH, of bone). Absence of sclerosis made pyogenic spondylitis unlikely.

Unifocal LCH is a rare disease which can present as collapse of a single cervical vertebra in a child [3,13]. Diagnosis of unifocal LCH could be confirmed only by histology. Normal bone marrow, absence of osteolytic lesions in other bones and lack of ultrasonic evidence of liver or spleen involvement excluded multisystem histiocytosis.

The incidental finding of a focal calcified nodule in her liver further supported TB. TB is the most likely cause for asymptomatic calcified hepatic nodule in Sri Lanka because parasitic infections such as schistosomiasis or visceral leishmaniasis are extremely rare. A biopsy of liver nodule was not undertaken because indications needed to rule out underlying liver disease or diagnose a treatable parasitic infection were not present.

Anti-TB treatment with quadruple regime was administered until prevertebral soft tissue collection resolved. Although the liver is an uncommon site for calcified tuberculous nodule its disappearance after anti-TB treatment was significant. A full treatment course of 12 months ended with clinical and radiological evidence of healing with no neurological deficit or deformity [14].

## Conclusions

The primary diagnosis of spinal TB in our patient was based on a combination of clinical (night cries, restless sleep, long-standing pain and stiffness), radiological (‘cold abscess’ on MRI) and nonspecific laboratory findings (raised ESR, lymphocytosis). The learning points in this case report are the need for a high index of suspicion, value of a careful history and how a combination of nonspecific findings including the incidental finding of a calcified hepatic nodule helped in diagnosing spinal TB even when at a very unusual site.

## Consent

Written informed consent was obtained from the patient’s legal guardians, her parents, for publication of this case report and the accompanying three images. A copy of the written consent is available for review by the Editor-in-Chief of this journal.

## Abbreviations

BCG: Bacillus Calmette–Guérin; C3: Third cervical vertebra; ESR: Erythrocyte sedimentation rate; LCH: Langerhan cell histiocytosis; MRI: Magnetic resonance imaging; TB: Tuberculosis.

## Competing interests

The authors declare that they have no competing interests.

## Authors’ contributions

MS was responsible for the clinical care and diagnostic workup of this patient, and drafted the manuscript. IK was involved in providing clinical management and participated in writing the manuscript. Both authors have intellectually contributed to this article. Both authors read and approved the final manuscript.
